# Real-World Use of GLP-1 Receptor Agonist Liraglutide in Adolescents with Obesity: A First Longitudinal Single-Center Analysis from Switzerland †

**DOI:** 10.3390/children12121716

**Published:** 2025-12-18

**Authors:** Cees Noordam, Urs Eiholzer, Claudia Katschnig, Aikaterini Stasinaki, Ilja Dubinski

**Affiliations:** 1 Center for Paediatric Endocrinology Zurich (PEZZ), Moehrlistrasse 69, 8006 Zurich, Switzerland; 2 Division of Paediatric Endocrinology and Diabetology, Dr. von Hauner Children’s Hospital, Ludwig Maximilian University Munich (LMU), 80337 Munich, Germany

**Keywords:** adolescent obesity, Glucagon-like Peptide-1, Glucagon-like Peptide-1 (GLP-1) receptor agonists, liraglutide, Saxenda, body mass index

## Abstract

**Highlights:**

**What are the main findings?**

• Liraglutide reduced clinically relevant BMI-SDS in adolescents with obesity under real-world clinical conditions, with a safety profile consistent with clinical trials.
• Despite efficacy, treatment discontinuation was common, mainly due to limited weight loss or mild nausea.

**What are the implications of the main findings?**

• Thorough pre-treatment counseling is essential to set realistic expectations and address manageable side effects.
• Further studies are needed to identify factors that may predict a favorable response to therapy, and to clarify the broader role of GLP-1 receptor agonists in adolescent obesity.

**Abstract:**

**Background:** Adolescent obesity remains a challenge with limited treatment options. GLP-1 receptor agonists such as liraglutide (Saxenda^®^) have shown efficacy in trials, but real-world data in youth are scarce. **Methods:** This retrospective longitudinal, non-interventional study analyzed 22 adolescents treated with liraglutide in a Swiss pediatric endocrinology center. All received non-structured nutritional/lifestyle counseling with three-monthly follow-up. BMI standard deviation scores (BMI-SDS) and adverse effects were recorded. **Results:** The mean age at initiation was 14.9 years (range 12.5–17.5); 15 patients had Southern European immigrant background. Mean treatment duration was 8.2 months (range 1–18). BMI-SDS decreased significantly from +2.63 (IQR +2.4/+2.8) to +2.40 (IQR +2.2/+2.6). Median intra-individual reduction was −0.20 (IQR −0.28/−0.10), *p* = 0.0003 with large effect size (rb = −0.77). Thirteen patients discontinued treatment, mainly due to insufficient weight loss or mild nausea. In the patients continuing therapy BMI-SDS decreased from +2.59 (IQR +2.4/+2.8) to +2.08 (IQR +1.9/+2.4). No serious adverse events occurred. **Conclusions:** Liraglutide showed a comparable efficacy to the pivotal clinical trial in reducing BMI-SDS in adolescents, while the side-effect profile was similarly mild and consistent with previously reported data. Discontinuation rates remained high, highlighting the need for thorough pre-treatment counseling on expected mild, transient symptoms and strategies to mitigate nausea. Future prospective studies are needed to assess long-term outcomes and identify patient characteristics associated with greater treatment success, to better individualize GLP-1–based therapy in adolescent obesity.

## 1. Introduction

Obesity is a health condition that is associated with numerous metabolic and psychological complications in the long term [1,2]. While the prevalence of overweight and obesity among children and adolescents in high-income countries has stabilized at a high level, it is on the rise in low-income countries [3,4]. The stabilization or even a slight decrease in this trend has recently been demonstrated for Switzerland [5]. Moreover, the leading role of migration in the increase in overweight and obesity has also been demonstrated [6]. Children with parents from Southern European countries have a 2.5-fold higher risk of being overweight in Switzerland [6,7].

Obesity is increasingly recognized as a complex, multifactorial chronic disease that arises from the interplay of biological, behavioral, environmental, and psychosocial determinants, rather than from a single causal pathway [8]. The treatment of adolescents with overweight and obesity places high demands on all those involved and is often accompanied by little success, also with regard to lasting changes [8]. Although lifestyle-based interventions typically achieve only modest reductions in body weight, their overall effectiveness remains limited [1]. The use of GLP-1 (Glucagon-like Peptide-1) receptor agonists (e.g., Liraglutide and Semaglutide) together with lifestyle interventions has shown greater weight loss than conventional non-pharmacological treatment [9,10,11,12]. GLP-1 receptor agonists reduce body weight primarily by activating hypothalamic satiety pathways, delaying gastric emptying, suppressing glucagon secretion, and lowering food intake, thereby improving both energy balance and metabolic control [13]. Whether metabolic parameters can also be improved in children and adolescents is the subject of current studies [14]. However, the side effect profile in children and adolescents appears to be largely favorable and mainly related to nausea [14,15,16]. Indications of an increased risk of suicidal behavior have not been confirmed to date [17,18]. The data available for children under the age of 12 is very limited but also shows efficacy in terms of reducing weight and waist circumference [19,20]. The prescription rate has increased significantly in children and adolescents in the United States, although clear ethnic differences have been observed [21].

In Switzerland, both Saxenda^®^ (Liraglutide, since June 2023) and Wegovy^®^ (Semaglutide, since May 2025) are reimbursed for adolescents ≥12 years with ≥60 kg body weight and obesity defined by IOTF [22] age- and sex-specific BMI thresholds equivalent to an adult BMI ≥ 35 kg/m^2^ [23,24]. Prescription of either drug is restricted to pediatric endocrinologists or certified pediatric obesity centers and requires prior insurer approval, documented calorie restriction, structured physical activity, and three-monthly follow-up. Wegovy^®^ must be discontinued if BMI or BMI z-score does not improve by ≥5% after 28 weeks [23], whereas Saxenda^®^ must be stopped if BMI fails to improve by ≥4% after 12 weeks at the maximal tolerated dose [24].

In this retrospective longitudinal, non-interventional real-life data study, we present outcomes from 22 adolescents treated with Saxenda^®^. Based on the pivotal trials, we anticipated a comparable reduction in BMI. In the SCALE Kids trial by Fox et al. [9], children aged 6 to <12 years with obesity showed a mean BMI reduction of −5.8% at 56 weeks compared to placebo. Similarly, in the adolescent trial by Kelly et al. [10], Liraglutide resulted in a mean BMI-SDS reduction of −0.22 after 56 weeks.

## 2. Materials and Methods

### 2.1. Patient Characteristics

The first patients initiated treatment in autumn 2023. To date, a total of 22 adolescents have been treated, comprising 13 males and 9 females. Patients were not actively selected; all individuals referred by their primary care physicians and willing to initiate treatment after detailed counseling were included. The mean age at treatment initiation was 14.9 years, ranging from 12.5 to 17.5 years. None of the patients had manifest type 2 diabetes mellitus. Notably, the study population included a high proportion of children with a Southern European immigrant background (15 out of 22), whereas only seven were of Swiss origin, four of whom belonged to the Jewish Orthodox community. The ethnic composition of the cohort reflects the higher prevalence of obesity among adolescents with Southern European migration background in Switzerland and is consistent with previously published population-based data [6,7]. All patients received nutritional counseling and advice on lifestyle changes with regard to diet and exercise. Instead of a formalized program, counseling was incorporated into the regular outpatient visits, with about 15 min devoted to nutritional and lifestyle advice during each consultation provided by the pediatric endocrinologist. All patients were seen three-monthly for clinical visits. Parents or caregivers were trained in managing therapy and potential side effects, while responsibilities for day-to-day therapy monitoring were managed within the family.

### 2.2. Treatment and Observational Details

Treatment duration averaged 8.2 months (range 1–18). All participants were treated with liraglutide (Saxenda^®^), which was the only approved pharmacologic option for adolescents at that time.

No patient who received liraglutide was excluded. In accordance with the manufacturer’s recommendations, Saxenda^®^ dose escalation was performed stepwise, starting with 0.6 mg once daily for the first week, followed by 1.2 mg, 1.8 mg, 2.4 mg, and finally the maintenance dose of 3.0 mg once daily. If the next dose level was not tolerated, the treatment was continued at the previously tolerated dose; doses above 3.0 mg were not used as they are not recommended.

Adherence was not formally assessed; however, controlled medication dispensing allowed estimation of treatment adherence based on documented drug supply.

All patients received Saxenda^®^, as it was the only GLP-1 receptor medication approved for use in adolescents at that time. From the patient files were retrieved: sex and ethnicity; age, height, weight and BMI at start of therapy; treatment duration, discontinuation before end of observation period yes/no, reason for discontinuing therapy, and height, weight and BMI at the end of therapy or at the last visit in patients continuing therapy.

Treatment success was not formally defined for this study; however, we report the number of patients fulfilling the insurance company’s mandatory response criteria at 4 months, which represent the only externally defined threshold for continuation in our setting. According to Swiss prescribing guidelines, Saxenda treatment is continued only if BMI or BMI z-score improves by ≥4% after four months of therapy; it is indicated for adolescents ≥ 12 years with obesity defined by age- and sex-specific BMI thresholds according to the International Obesity Task Force (IOTF). Drop-out was defined as the moment at which liraglutide therapy was discontinued, which in all but two cases was initiated by the patient.

Thus, in the absence of universally accepted, clinically validated response criteria for GLP-1 receptor agonist therapy in adolescents, no predefined definition of treatment success was applied in this real-world study. BMI-SDS change was therefore used as a widely accepted surrogate marker of treatment response.

### 2.3. Statistical Methods

All statistical analyses were conducted using R version 4.5.1 [25]. The primary outcome variable was the body mass index (BMI), normalized for age and sex, called BMI standard deviation score (BMI-SDS). As the baseline BMI-SDS exhibited a non-normal distribution (via histogram, Q–Q plot, and Shapiro–Wilk test *p* = 0.06), a nonparametric approach was adopted to compare paired observations. Descriptives are given as median, inter quartile range (IQR) and total range of values. To assess whether there was a statistically significant change in BMI over time, the Wilcoxon signed-rank test was used to compare BMI-SDS at baseline and at the last visit available or at the visit where the patient discontinued therapy, respectively. To quantify the effect size, the rank-biserial correlation (r_b_) was calculated. This measure is suitable for paired nonparametric tests and reflects the degree of separation between the two related samples on a rank scale [26]. All tests were two-tailed with a significance threshold set at *p* < 0.05. The 95% Confidence Interval (CI) for r_b_ was computed using bootstrapping with 1000 resamples.

## 3. Results

**Figure 1:**
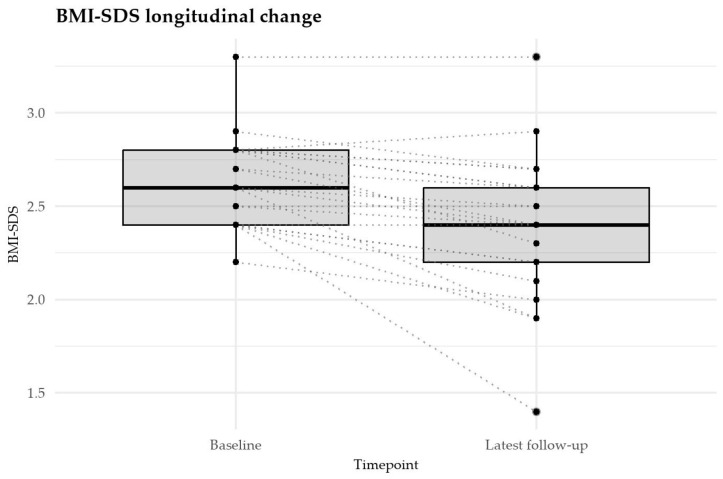
Longitudinal change in BMI-SDS from baseline to latest follow-up. Boxplots depict the median, interquartile range, and range of BMI-SDS values. Individual trajectories are shown as dotted lines connecting paired measurements for each participant.

In all but three patients the dose was titrated up to 3 mg; in one patient the maximum dose reached was 1.8 mg, and in two patients the maximum dose was 2.4 mg. The average treatment duration was 8.2 months, with a range of 1 to 18 months. The median BMI-SDS decreased from +2.63 (IQR +2.4 to +2.8, total range +2.2 to +3.3) at treatment initiation to +2.4 (IQR +2.2 to 2.6, total range +1.9 to +3.3) at the last follow-up (Figure 1). The median intra-individual reduction in BMI-SDS was −0.20 SDS (IQR −0.28 to −0.10, full range −1.0 to 0.1), which was highly significant (Wilcoxon signed-rank test *p* = 0.0003) with a large effect size (r_b_ = −0.77). The 95% CI for r_b_ was −0.86 to −0.63.

In the patients continuing therapy (9 out of 22 patients, representing 41%), BMI-SDS decreased from +2.59 (IQR +2.4 to +2.8, total range +2.2 to +2.9) to +2.08 (IQR +1.9 to +2.4, total range +1.4 to +2.7). Thus, in those continuing treatment, the median intra-individual reduction in BMI-SDS was −0.51 SDS.

In practical terms, a reduction of −0.5 SDS in body weight can represent a clinically meaningful change. For instance, Patient 19 in our cohort initially presented with a body weight of 105.8 kg. After approximately six months, her weight had decreased to 85.8 kg, corresponding to an absolute loss of 20.0 kg. This substantial reduction is reflected in the SDS metric as a shift of −0.5, underscoring how even a seemingly moderate change in standard deviation units may translate into a pronounced and tangible improvement in individual patients.

However, Saxenda^®^ was discontinued in 13 patients for different reasons (Table 1). No serious adverse events were reported. In the patients who discontinued therapy, BMI-SDS changed from +2.63 (IQR +2.4 to +2.8, total range +2.4 to +3.3) at baseline to +2.58 (IQR +2.4 to +2.7, total range +2.1 to +3.3) at the last visit. There were no significant differences in age, baseline BMI-SDS, or sex distribution between patients who continued treatment (mean age 14.8 years, mean BMI-SDS 2.59, sex ratio F/M = 4/5) and those who discontinued therapy (mean age 15.0 years, mean BMI-SDS 2.65, sex ratio F/M = 5/8). Those who discontinued received therapy for a mean of 7.6 months (range 1–15 months), while patients who remained in treatment were on therapy for a mean of 9.7 months (range 4–18 months).

Regarding the Swiss reimbursement criterion of a ≥4% BMI reduction at 4 months, 20 patients completed at least four months of therapy, of whom 19 met the criterion and one did not.

## 4. Discussion

In this real-life cohort, we were able to show the effectiveness of GLP-1 receptor agonist therapy with Liraglutide/Saxenda^®^ in adolescents in combination with lifestyle counseling. The efficacy, measured by the intraindividual change in BMI-SDS before treatment and at the time of evaluation (last follow-up visit), corresponds to the published results from the pivotal phase 3 trial [10]. Although the observed BMI-SDS reduction is numerically similar to that reported by Kelly et al., differences in study design—including the absence of a placebo control—as well as the wide range of treatment durations and the shorter follow-up compared with the pivotal 56-week trial limit direct comparability and the assessment of long-term efficacy [10]. In those continuing treatment, the reduction in BMI-SDS was −0.51 SDS. Our data allow for this distinction, which has not been clearly differentiated in previously reported cohorts.

In contrast, the Spanish real-world study reported a substantially greater mean BMI-z reduction of −1.09 ± 0.24 after a similar treatment duration, likely reflecting the combination with intensive lifestyle interventions and the higher baseline obesity severity in their cohort [27]. Although intensive lifestyle interventions represent the recommended standard of care, they are not uniformly accessible or sustainable in routine clinical practice, which this real-world study was designed to reflect. This finding also highlights the considerable interindividual variability in treatment response and emphasizes the need to identify clinical or behavioral predictors of good response. The greater efficacy reported in the Spanish study may in part relate to a higher concentration of such responders within their intensively managed cohort.

However, our observation period is shorter than the 56 weeks in the Kelly et al. study [10], meaning that persistent weight losses are possible. In contrast to the marked BMI-z reduction in the Spanish cohort, the single-center Qatari study by Dauleh et al. found no significant changes in BMI or BMI-SDS after 72 weeks among the 12 patients who completed treatment (45% drop-out rate) [28]. The Slovenian real-world study reported a modest six-month BMI-SDS reduction (−0.28 SDS), comparable to our findings [29]. In the Austrian HBLT study, adolescents treated with liraglutide in combination with structured lifestyle therapy showed a mean BMI reduction of −2.7% (−6.24% among continuers). This is comparable to the modest BMI-SDS decrease observed in our cohort, though direct comparison is limited by differing outcome measures and study designs [30].

Moreover, obesity research is complicated by placebo and nocebo effects [31], and the Hawthorne effect may similarly influence weight-loss motivation during scheduled follow-ups [32], although these factors remain insufficiently explored in adolescents. Structured pediatric obesity programs (without GLP-1 receptor agonists) are expensive and yield only limited weight reduction, which restricts their practicality in everyday healthcare [33]. In our cohort, lifestyle counseling—including nutritional guidance—was therefore not delivered through a structured program but as an individualized multicomponent approach integrated into routine clinical consultations by the pediatric endocrinologist. While this introduces variability in counseling intensity, it reflects the real-world clinical setting in which approaches to lifestyle counseling may differ across centers and practitioners.

With regard to the side effect profile, the typical symptoms of nausea were observed. No other new symptoms or serious unexpected side effects occurred during the observation period. In four of our 22 patients, nausea led to discontinuation of treatment (approx. 23% of study participants), while mild nausea symptoms were described in 64.8% of Kelly et al. [10]. Gastrointestinal side effects were also the most common in the study by Danne et al. [16,28].

However, the proportion of treatment discontinuations is remarkably high, underscoring the need for thorough pre-treatment counseling to inform patients about expected, generally mild symptoms and their often-transient nature. Furthermore, general measures to reduce nausea should always be integrated into treatment information and initiation (e.g., advice on eating habits, food composition and lifestyle) [34]. This includes eating smaller portions more slowly, reducing fatty foods and stopping eating when satiated. Our cohort showed a dropout rate of 59% (13 of 22 patients), which is higher than the 41% reported in the Austrian HBLT study [30] and the ~45% observed in the Qatari cohort [28]. This aligns with findings in adults, where discontinuation rates of GLP-1 receptor agonists reached 50.3% [35]. The factors underlying treatment adherence versus discontinuation in adolescents remain unclear and warrant further investigation.

Regarding the Swiss reimbursement criterion of a ≥4% BMI reduction at 4 months, 19 of 22 patients met this requirement; only three failed to reach the threshold, two of whom discontinued treatment before three months—highlighting that treatment success depended more on persistence than on reimbursement-related restrictions. While the ≥4% BMI reduction threshold is administrative, BMI-SDS reduction remains a clinically relevant surrogate associated with improved health trajectories in adolescents with obesity.

Notably, the ethnic composition of our cohort does not reflect the general demographic distribution in Switzerland and at our center but is skewed towards adolescents with Southern European roots (68%). This pattern aligns with previously reported disparities in the prevalence of overweight and obesity within Swiss society [5,6,7,35]. Understanding the demographic profiles associated with current GLP-1 receptor agonist prescribing patterns may support the development of more tailored therapeutic guidance and follow-up [21]. Importantly, previous work has shown that the disproportionately high prevalence of obesity among children of Southern European origin in Switzerland is largely genetically determined rather than explained by cultural, socio-economic, or dietary factors [6].

This study is limited by its small sample size and retrospective design, which restrict the strength and generalizability of the findings. The absence of predefined criteria for treatment success and the heterogeneity in treatment duration introduce additional variability.

From our point of view, it would be worthwhile to study the pre-therapy factors that could correlate with treatment effectiveness. These could potentially include personality types, cognitive characteristics, demographic and ethnic factors, social and environmental factors or genetic aspects. This requires well-designed prospective studies with a diverse patient population and longer follow-up [36]. Other GLP-1 receptor agonists, such as Semaglutide/Wegovy^®^ [11], could have better effects in adolescents [37]. Identifying predictors of treatment response will be key to optimizing and personalizing GLP-1 receptor agonist therapy in this population.

## 5. Conclusions

In summary, treatment with GLP-1 receptor agonists in our cohort was effective in 41% of the adolescents and the side-effect profile was consistent with expectations, albeit with a high drop-out rate. In our setting, lifestyle counseling was not delivered within a structured program but provided as a component of routine visits by the attending pediatric endocrinologist. Further evidence is needed to support broader use in this population, and in the meantime, emphasis should be placed on addressing the reasons for discontinuation through clear pre-treatment counseling and strategies to mitigate nausea.

## Figures and Tables

**Table 1:** Drop-out reasons and doses in 13 individual patients.

Individual Patient	Dose at Drop-Out	Reason for Drop-Out
Patient 1	3.0 mg	Patient’s wish, not specified
Patient 2	3.0 mg	Not happy with result
Patient 3	3.0 mg	Painful injections
Patient 4	3.0 mg	Tiredness, wishes bariatric operation
Patient 5	3.0 mg	Did not comply with insurance
Patient 6	3.0 mg	Nausea
Patient 7	2.4 mg	Nausea
Patient 8	3.0 mg	Not happy with result
Patient 9	3.0 mg	Nausea
Patient 10	3.0 mg	Nausea
Patient 11	3.0 mg	Patient’s wish, not specified
Patient 12	3.0 mg	Patient’s wish, not specified
Patient 13	3.0 mg	Did not comply with insurance

## Data Availability

The data presented in this study are available on request from the corresponding author. The data are not publicly available due to privacy and ethical reasons. We also wish to disclose that an abstract of this work was presented as a poster (No. 77) at the Annual Meeting of the Swiss Endocrinology Society in Lucerne, Switzerland, on 14 November 2025 [38].

## Abbreviations

The following abbreviations are used in this manuscript:

BMI	Body Mass Index
GLP-1	Glucagon-like Peptide-1
SDS	Standard Deviation Score
